# Historical trends in teacher personality from human language

**DOI:** 10.1073/pnas.2413253121

**Published:** 2024-10-08

**Authors:** Liang Xu

**Affiliations:** ^a^Department of Psychology, College of Education, Zhejiang University of Technology, Hangzhou 310014, China; ^b^Department of Psychology and Behavioral Sciences, Zhejiang University, Hangzhou 310058, China

**Keywords:** teacher personality, Big Five personality, historical trend, Big data analysis, Google books

## Abstract

Understanding the historical perception and value of teacher personalities reveals key educational priorities and societal expectations. This study analyzes the evolution of teachers’ ascribed Big Five personality traits from 1800 to 2019, drawing on millions of English-language books. Word frequency analysis reveals that conscientiousness is the most frequently discussed trait, followed by agreeableness, openness, extraversion, and neuroticism. This pattern underscores society’s focus on whether teachers are responsible. Polarity analysis further indicates a higher prevalence of low neuroticism descriptors (e.g., patient and tolerant) in descriptions of teachers compared to the general population, reinforcing the perception of teachers as stable and dependable. The frequent use of terms like “moral”, “enthusiastic”, and “practical” in describing teachers highlights the positive portrayal of their personalities. However, since the mid-20th century, there has been a notable rise in negative descriptors related to openness (e.g., traditional and conventional), coupled with a decline in positive openness terms. This shift suggests an evolving view of teachers as less receptive to new ideas. These findings offer valuable insights into the historical portrayal and societal values attributed to teacher personalities.

Teachers play a pivotal role in shaping students’ educational and developmental trajectories (1). Understanding historical perceptions and the perceived importance of different personality traits in teachers offers essential insights into educational priorities and societal expectations. The Big Five personality traits (agreeableness, extraversion, conscientiousness, neuroticism, and openness) significantly influence teaching styles, classroom management, and student outcomes (2, 3). However, there is a notable lack of research on the evolution of these traits’ perceptions in teachers over time.

The lexical hypothesis of the Big Five model suggests that socially relevant and frequently used descriptors are preserved in natural language (4, 5). Analyzing human records with personality descriptions can effectively outline the traits attributed to different groups (6, 7), capturing both actual perceptions and ideal expectations within societal contexts (8). Additionally, as Berger and Luckmann have noted, language and social change are deeply interconnected (9), suggesting that historical shifts may have also impacted how teachers’ personalities are described. For instance, previous studies have used word frequency analysis in large-scale corpora to uncover generational changes in the personality descriptions of the general population (10) and differences across gender and age groups (8). Consequently, examining linguistic data can also provide valuable insights into the historical evolution of the personality traits ascribed to teachers.

This study analyzes the adjusted usage frequency of personality words in 16,513,571 English-language books (11) to address two primary questions: Which Big Five personality dimensions were most frequently emphasized for teachers and whether there has been a shift in focus over time, and how personality descriptions of teachers differ from those of the general population.

## Results

This study first compared the total adjusted frequency of different Big Five personality words used to describe teachers from 1800 to 2019. The Friedman test reveals significant differences in the use of these words for describing teachers (*χ^2^*(4) = 741.553, *P* < 0.001). Conscientiousness was the most frequently noted trait (all *P* < 0.001, effect size *r* > 0.356), followed by agreeableness, openness, extraversion, and neuroticism. Compared to the general population, teachers’ agreeableness, extraversion, and conscientiousness received more attention in the 19th century (all *P* < 0.001, effect size *r* > 0.793). However, this trend reversed in the 20th century and persisted into the 21st century due to declining descriptions of teachers’ agreeableness [Sen’s slope (SS) = −1.474 × 10^−5^], extraversion (SS = −8.173 × 10^−6^), and conscientiousness (SS = −8.058 × 10^−5^). These findings reveal which personality dimensions of teachers have been more prominently discussed during different historical periods.

**Fig. 1.**
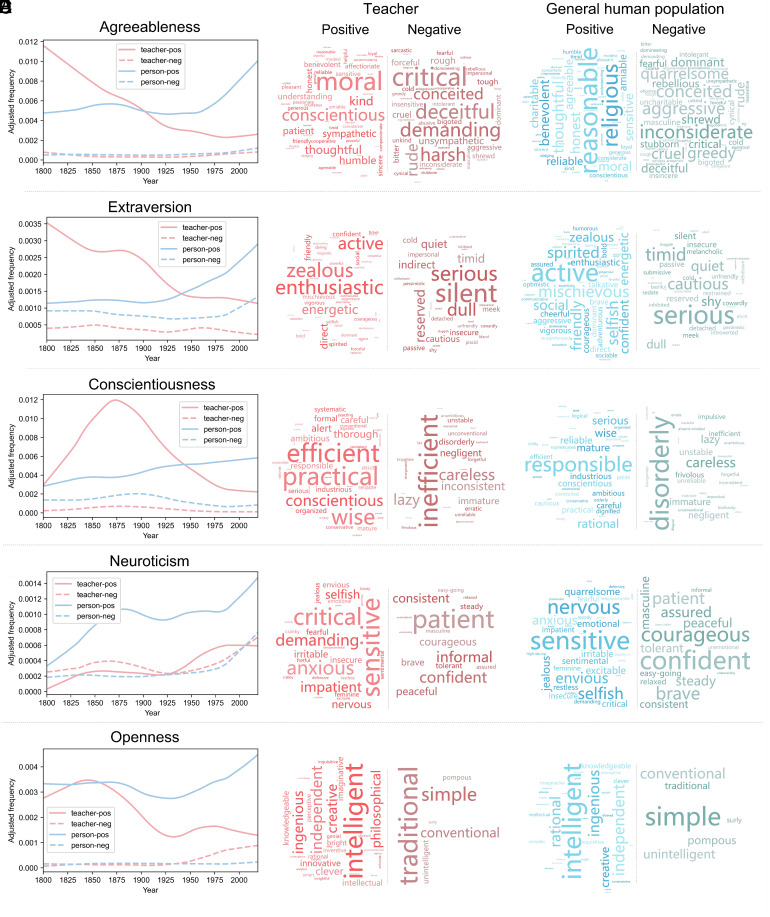
Historical trends in polarity of big five personality factors when describing teachers and the general human population (1800 to 2019). Panels *A*–*E*, respectively, depict the historical trends in polarity (positive and negative) for each Big Five personality factor—agreeableness, extraversion, conscientiousness, neuroticism, and openness—from 1800 to 2019 when describing teachers and the general human population (loose smoothing), along with word clouds of the most frequently used terms. “teacher-pos” and “teacher-neg” indicate positive and negative personality words used to describe teachers, respectively; while “person-pos” and “person-neg” represent positive and negative personality words used to describe the general human population.

The subsequent polarity (positive and negative) analysis revealed several important trends (Fig. 1). For example, the frequency of negative words related to openness (e.g., traditional and conventional) used to describe teachers increased steadily from the mid-20th century, eventually surpassing their use for the general population (Z = 7.220, *P* < 0.001, effect size *r* = 0.869). This substantial rise, combined with the decline in positive openness words, indicates a shift in societal perception toward decreasing openness in teachers over time. Additionally, although adjectives for many personality dimensions are used more frequently to describe the general population, words negatively loaded in the neuroticism factor (e.g., patient and tolerant) are more commonly used to describe teachers (Z = 7.028, *P* < 0.001, effect size *r* = 0.474). Moreover, the word cloud patterns align with Zipf’s Law of word frequencies (a few words account for most of the word occurrences) (12), highlighting the primary descriptors of teacher personality (such as moral, enthusiastic, practical, and patient). These findings indicate significant changes in the language used to describe teacher personalities over time.

## Discussion

This study uses word frequency analysis of extensive human records to map the historical profile of teachers’ Big Five personality traits. Conscientiousness is the most emphasized trait, underscoring the educational priority on diligence, reliability, and responsibility in teaching (13). Furthermore, compared to the general population, the higher frequency of low neuroticism descriptors (e.g., patient and tolerant) in teachers underscores the value placed on emotional stability in teaching—a profession characterized by diverse and often challenging classroom dynamics. Emotional resilience and calmness are likely seen as essential for maintaining a positive learning environment. Additionally, the recurrent use of positive descriptors like “moral”, “enthusiastic”, and “practical” underscores the perception of teachers as ethical role models who not only impart knowledge but also guide students in their personal development. These attributes are considered essential for inspiring students and facilitating their holistic growth, both academically and morally.

However, the study also observes an increased use of negative descriptors related to openness (e.g., traditional and conventional) since the mid-20th century, alongside a decline in positive openness words. This shift notably coincides with the post-World War II era, suggesting that in times of peace, there is heightened societal attention on educational development. The rise in negative descriptors may reflect growing dissatisfaction with perceived rigidity in education (14), as well as an increasing demand for educators to be more adaptable and innovative in response to evolving societal expectations. In summary, these findings reveal significant historical shifts in how teacher personalities are viewed and valued.

While analyzing the world’s largest corpus offers valuable insights, it is subject to certain systematic biases, as noted in previous work (15). These include variations in book inclusion policies over time (e.g., editorial practices, the proportion of certain genres, and translations), the exclusion of nonbook materials, and semantic drift. Moreover, focusing solely on English-language books confines the study’s findings to English-speaking cultures. Additionally, while the used dictionary approach provides clear and interpretable results, it may not capture negations; combining it with topic modeling and other NLP methods in future research could improve the reliability of the findings.

## Materials and Methods

This study analyzed over 16 million books from the Google Books Ngram database (9) from 1800 to 2019. Using word frequency methodology, I calculated adjusted frequencies for 435 personality adjectives (4) describing *teacher/teachers* and *person/persons* over time. The Friedman test, Wilcoxon signed-rank test, and Sen’s slope analysis were used to compare and assess trends in the Big Five personality dimensions and their polarities for teachers and the general population. Supplementary analyses for fiction and nonfiction books, as well as different targets (professor and student), were conducted to strengthen the study’s validity. Methodological details are provided in *SI Appendix*. Detailed process data and codes can be found at the OSF Repository (https://osf.io/3ne64/).

## Supplementary Material

Appendix 01 (PDF)

## Data Availability

Excel spreadsheets (.xlsx), SPSS data files (.sav and .spv), Python scripts (.py). data have been deposited in The Open Science Framework (OSF) (https://osf.io/3ne64/) (16). Previously published data were used for this work (Google Books N-gram dataset: https://storage.googleapis.com/books/ngrams/books/datasetsv3.html) (17).
