# Supplementary material for: Historical trends in teacher personality from human language
Source: Proc Natl Acad Sci U S A. 2024 Oct 8;121(42):e2413253121. doi: 10.1073/pnas.2413253121 (PMC11494301; doi:10.1073/pnas.2413253121)
Supplement: Supplementary file 1 — Appendix 01 (PDF) [file pnas.2413253121.sapp.pdf]

Supporting Information for

Historical Trends in Teacher Personality from Human Language

Liang Xu[1,2]

1. Department of Psychology, College of Education, Zhejiang University of Technology, Hangzhou 310014, China.

2. Department of Psychology and Behavioral Sciences, Zhejiang University, Hangzhou 310058, China.

* Liang Xu.

Email: xuliang_psy@zju.edu.cn

This PDF file includes:

Supporting Information Text
SI References

Supporting Information Text

Methods

1. Data description

This study utilized the English-language corpus from the third iteration of the Google Books Ngram (GBN) database, accessible at GBN Dataset (http://storage.googleapis.com/books/ngrams/books/datasetsv3.html). The dataset is derived from a diverse array of sources, including university libraries and publishers, and encompasses over 16 million English books (1). Due to the limited availability of books published before 1800, this research focuses on the period from 1800 to the most recent data point in 2019. Consequently, a detailed analysis was conducted on a total of 16,513,571 English-language books published between 1800 and 2019. The process data and codes are available at OSF Repository (https://osf.io/3ne64/).

2. Word frequency calculation

Goldberg (1993) emphasized that the frequency of word usage can serve as a valuable indicator of the importance attributed to specific personality traits (2). Descriptors of more salient characteristics are more prevalent within large corpora (3). Building on previous research (4,5), this study employed usage frequency as the primary metric for analysis. The initial step involved conducting targeted searches to determine the frequencies of combinations of "personality adjectives" and "target" in the Google Books English corpus (e.g., *traditional teacher* and *reliable person*). In this study, "personality adjectives" refers to a set of 435 adjectives identified by Saucier and Goldberg (6), while "target" includes the terms *teacher/teachers* and *person/persons*.

To account for potential variations in the usage frequencies of the "target" term across different time periods, we normalized the frequencies of adjective-target combinations, following previous work (7). This normalization was achieved by dividing the frequencies of these combinations by the usage frequencies of the respective "target" term, thereby yielding adjusted frequencies for each adjective-target pairing. For example, the adjusted frequency of *traditional teacher* in year 2000 is calculated as follows:

$$Adjusted\ frequency_{2000}^{traditional\ teacher} = \frac{Usage\ frequency_{2000}^{traditional\ teacher}}{Usage\ frequency_{2000}^{teacher}},$$

where $Usage\ frequency_{2000}^{traditional\ teacher}$ is the usage frequency of *traditional teacher* in year 2000 and $Usage\ frequency_{2000}^{teacher}$ is the usage frequency of *teacher* in year 2000.

3. Analysis strategy

3.1 Calculating the total adjusted frequency per year for each big five personality dimension

The primary objective of this study was to identify which dimensions of the Big Five personality traits were most frequently emphasized in descriptions of teachers and to assess any temporal shifts in these emphases. To achieve this, personality adjectives were categorized under one or more personality factors based on a threshold of 0.30 (7,8), as grounded in their factor loadings in the work of Saucier and Goldberg (6). For instance, the adjective "traditional" was classified under the factor of openness due to its loadings of 0.14 on agreeableness, -0.14 on extraversion, 0.28 on conscientiousness, 0.02 on neuroticism, and -0.36 on openness. Next, the adjusted frequencies of all adjectives corresponding to each Big Five factor were aggregated annually, yielding the total adjusted frequency for each personality factor per year.

3.2 Comparing the total adjusted frequency for each big five personality dimension in descriptions of teachers

To compare the total adjusted frequency per year for each Big Five personality dimension in descriptions of teachers from 1800 to 2019, the Friedman test was employed. This test assessed whether significant differences existed among the five dimensions. Subsequently, post hoc Wilcoxon tests were conducted for pairwise comparisons of the five dimensions. The process data and codes used for this analysis are available at the OSF Repository (https://osf.io/3ne64/; Step 1).

In addition, I also reported the traditional effect size *r* ($r = \frac{Z}{\sqrt{N}}$; *Z* is the test statistic and *N* is the total number of observations) from the post hoc Wilcoxon tests to highlight the magnitude of the difference between the ranks of two personality traits. For example, the main manuscript states, "Conscientiousness was the most frequently noted trait (all $p < 0.001$, effect size $r > 0.356$), followed by agreeableness, openness, extraversion, and neuroticism." This indicates that even in the weakest comparison, conscientiousness had an effect size greater than 0.356, reflecting a significantly higher frequency of descriptions related to conscientiousness compared to other traits. This underscores the societal emphasis on the importance of conscientiousness in teachers. In addition, presenting both this traditional effect size metric r (9) and the natural metric (e.g., adjusted frequency related metric may be observed in Figure 1), suggested by the recent work (10), in the main manuscript may provide readers with a more comprehensive understanding of the findings.

3.3 Comparing differences in personality dimension descriptions between teachers and the general human population

This study also examined the differences in personality dimension descriptions between teachers and the general human population (GHP). To achieve this, the Wilcoxon signed-rank test was employed to compare the descriptions of the Big Five personality dimensions for teachers and the GHP across various time periods. For instances, the differences in the five dimensions were compared for the 19th century (Step 2), and the differences between teacher and GHP in the neuroticism and openness dimensions were compared from 1800 to 2019 (Step 4). These analyses correspond to the findings reported in the main manuscript and were included to validate and support the trends or differences described. Detailed process data and codes can be found at the OSF Repository (https://osf.io/3ne64/; Step 2, 4).

3.4 Assessing Trends in Personality Description Movements

To further describe the trends in each personality dimension over specific time periods, this study calculated Sen's slope for each personality dimension (11). A positive slope indicates an overall increasing trend, while a negative slope indicates a decreasing trend. For instance, Sen's slope was calculated for the five dimensions describing teachers from 1901 to 2000 to reflect their overall trends in the 20th century. These analyses correspond to the findings reported in the main manuscript and were included to validate and support the trends or differences described. Detailed process data and codes can be found at the OSF Repository (https://osf.io/3ne64/; Step 3).

3.5 Calculating the annual total adjusted frequency for the polarity (positive or negative) of each Big Five personality dimension

Sections 3.1 to 3.4 of the analysis primarily focused on the overall attention given to each personality dimension based on all adjectives associated with them. In fact, each personality adjective carries a polarity, indicating whether it is positively or negatively loaded within the personality dimension (6). Therefore, this study then conducted an analysis of the different polarities within each personality dimension. To achieve this, I aggregated the adjusted

frequencies of positive and negative words separately for each personality factor. For instance, under the extraversion factor, I computed the total adjusted frequency of positive descriptors such as *active*, *adventurous*, and *aggressive*. Conversely, I compiled the adjusted frequency of negative descriptors like *aloof*, *bashful*, and *cautious* to represent the negative polarity of extraversion.

3.6 Comparing the polarity of each personality dimension in describing teachers and the general human population

Similar to Section 3.3, the Wilcoxon signed-rank test was employed to compare the polarity of each personality dimension in describing teachers and the general human population (GHP) across various time periods. For instance, this study compared the frequency of negative words related to openness (e.g., *traditional* and *conventional*) used to describe teachers from 1951 to 2019 with those used for the GHP (Step 5); and assessed the differences in words negatively loaded in the neuroticism factor (e.g., *patient* and *tolerant*) between descriptions of teachers and the GHP from 1800 to 2019 (Step 6). These analyses correspond to the findings reported in the main manuscript and were included to validate and support the trends or differences described. Detailed process data and codes can be found at the OSF Repository (https://osf.io/3ne64/; Step 5, 6).

3.7 Loosing smoothing in trends presentation.

In addition, to visually depict changes in personality descriptions associated with each Big Five factor per year (see Figure 1 in main manuscript), I employed smoothing techniques using the *statsmodels* package in Python. This method involves applying loose smoothing to fit a curve to the dataset, enhancing clarity in trend illustration. The data were segmented into smaller subsets defined by a specified fraction of the total data (0.3 in this study), and polynomial regression was conducted within each subset. The regression weighting prioritizes proximity, assigning greater importance to data points closer in value and less to those more distant, utilizing a Gaussian kernel as the weighting function. This approach provides a refined visualization of temporal trends, effectively highlighting shifts in emphasis on different personality traits over time.

4 Supplementary analyses.
4.1 Supplementary analyses for fiction and nonfiction books.

Given that this study utilized the "English 2019" corpus from the GBN database, which inevitably contains 1,720,962 English fiction books (approximately 10.42% of the corpus), we conducted additional analyses to examine whether the high proportion of fiction books might influence the results. Specifically, we compared the analysis outcomes across fiction books, nonfiction books, and the complete "English 2019" corpus.

For fiction books, the analysis was directly based on the "English Fiction 2019" corpus within the GBN database. However, since the GBN does not provide a dedicated nonfiction corpus, the analysis of nonfiction books was performed by subtracting the "English Fiction 2019" data from the "English 2019" corpus. All other computational procedures followed the methods outlined in Sections 2-3 of the Supporting Information. Detailed process data and codes can be found at the OSF Repository (https://osf.io/3ne64/; Supplementary analyses for fiction and nonfiction books). I have also uploaded the code and data for plotting the visualization of trend changes (see “code for plotting personality trend changes in different books” and “data4pic”). Running this code will help you better observe the differences across various corpora.

The results for nonfiction books closely align with those of the "English 2019" corpus. The Pearson correlation coefficients between the whole English corpus ("English 2019") and the nonfiction books exceed 0.998 across all personality dimensions. For fiction books, the correlation between the whole corpus ("English 2019") and the fiction corpus ("English Fiction 2019") reaches a mean of 0.500±0.181, ranging from 0.205 (negative agreeableness words) to

0.836 (positive conscientiousness words). Although there are some differences in the absolute values of the adjusted frequency compared to the "English 2019" corpus, the rising and falling trends in adjusted frequency are very similar. These findings indicate that the results based on the "English 2019" corpus are reliable.

4.2 Supplementary analyses for student and professor.

To determine whether the trends in personality descriptions of teachers are general or specific to teachers, we compared these trends not only with the general human population but also with two other professions: students and professors. The analysis for students and professors followed the methods outlined in Sections 1-3 of the Supporting Information. Detailed process data for this part are available in the OSF Repository (https://osf.io/3ne64/; Supplementary analyses for student and professor). I have also uploaded the code and data for plotting the visualization of trend changes (see “code for plotting personality trend changes of different targets” and “data4pic”). Running this code will help you better observe the differences across different professions.

The result shows that the trends in personality descriptions differ across professions in most cases. This result indicates that the observed changes in the personality descriptions of teachers are, for the most part, specific to teachers.

Additionally, several interesting new findings emerged. For example, in the personality descriptions of students, words positively associated with neuroticism (e.g., anxious and sensitive) are more frequently used compared to words negatively associated with neuroticism (e.g., patient and peaceful), which is the opposite pattern observed for teachers and professors. We also observed intriguing coupling effects; for instance, starting in the mid-20th century, words negatively associated with openness (e.g., traditional and conventional) showed an increase in usage frequency for both students and teachers, while there was little change in the descriptions of professors. These findings provide important insights for future research on personality differences across professions.

SI References

1. J.-B. Michel, et al., Quantitative Analysis of Culture Using Millions of Digitized Books. *Science* 331, 176–182 (2011).
2. L. R. Goldberg, The structure of phenotypic personality traits. *Am. Psychol.* 48(1), 26–34 (1993).
3. D. Leising, J. Scharloth, O. Lohse, D. Wood, What Types of Terms Do People Use When Describing an Individual’s Personality? *Psychol. Sci.* 25, 1787–1794 (2014).
4. E. Roivainen, Frequency of the use of English personality adjectives: Implications for personality theory. *J. Res. Pers.* 47, 417–420 (2013).
5. E. Roivainen, Generational Changes in Personality: The Evidence from Corpus Linguistics. *Psychol. Rep.* 123, 325–340 (2020).
6. G. Saucier, L. R. Goldberg, Evidence for the Big Five in analyses of familiar English personality adjectives. *Eur. J. Pers.* 10, 61–77 (1996).
7. S. Ye, S. Cai, C. Chen, Q. Wan, X. Qian, How have males and females been described over the past two centuries? An analysis of Big-Five personality-related adjectives in the Google English Books. *J. Res. Pers.* 76, 6–16 (2018).
8. E. Roivainen, Personality Adjectives in Twitter Tweets and in the Google Books Corpus. An Analysis of the Facet Structure of the Openness Factor of Personality. *Curr. Psychol.* 34, 621–625 (2015).
9. J. Cohen, Statistical Power Analysis. *Curr. Dir. Psychol. Sci.* 1(3), 98–101 (1992).
10. F. M. Götz, S. D. Gosling, P. J. Rentfrow, Effect sizes and what to make of them. *Nat. Hum. Behav.* 8, 798–800 (2024).
11. P. K. Sen, Estimates of the Regression Coefficient Based on Kendall’s Tau. *J. Am. Stat. Assoc*. 63(324), 1379–1389 (1968).
